# Intake of iodine in a sample of UK mother–infant pairs, 6–12 months after birth: a cross-sectional study

**DOI:** 10.1017/S1368980025000230

**Published:** 2025-03-17

**Authors:** Jo Pearce, Jenny Christian, Lisa J Coneyworth

**Affiliations:** 1 Food & Nutrition, Sheffield Hallam University, Howard Street, Sheffield S1 1WB, UK; 2 Food, Nutrition & Dietetics, University of Nottingham, Sutton Bonington Campus, Sutton Bonington, Leicestershire, LE12 5RD, UK

**Keywords:** Iodine status, Infants, Complementary feeding, Dietary intake

## Abstract

**Objective:**

To investigate the intake of iodine in mother–infant pairs.

**Design:**

An exploratory, cross-sectional study. Iodine intake was estimated using Nutritics nutritional analysis software, following 24-h dietary recall. Iodine-rich foods were grouped and compared between those women who met the UK reference nutrient intake (RNI) for iodine (140 µg/d) and those who did not.

**Setting:**

Online and telephone questionnaires.

**Participants:**

Self-selecting caregivers of infants aged 6–12 months.

**Results:**

Ninety-one mother–infant pairs with a mean (sd) age of 33·2 (4·1) years and 8·4 (1·3) months, respectively, were included. Most mothers were exclusively breast-feeding (54·9 %). The estimated maternal median iodine intake from food and supplements (median 140·3 µg/d, just meeting the UK RNI for women of reproductive age, but not the World Health Organisation (WHO) or British Dietetic Association (BDA) recommendations for lactating women (250 µg/d and 200 µg/d, respectively). Forty-six (50·5 %) of mothers met the UK RNI. Estimated intakes of fish, eggs, cow’s milk and yoghurt/cream/dairy desserts were significantly greater, whilst intakes of plant-based milk alternative drinks were significantly less in mothers who met the RNI for iodine (*P* < 0·05) compared with those who did not. Infant iodine intake from food was positively correlated with maternal; total iodine intake, iodine intake from all food and iodine intake from dairy foods (Spearman’s rho = 0·243, 0·238, 0·264, respectively; *P* < 0·05).

**Conclusions:**

Women in the UK may not consume enough iodine to meet the demands of lactation. Guidance on iodine-containing foods, focussed on intake before and during pregnancy and lactation and mandatory fortification of plant-based milk-alternatives could all serve to avoid deficiency.

Mild-to-moderate iodine deficiency (ID) is an emerging problem in the UK, with younger women identified as particularly at risk^(1)^. Children <2 years of age are also acknowledged globally as being susceptible to ID^(2)^. Iodine is required to produce thyroid hormones which are critical for normal regulation of basal metabolic rate and metabolism. Iodine is also considered a crucial element in fetal programming, vital during the first 1000 days of life when infants and children require iodine for cognitive function, as well as growth and development^(3)^. ID in infancy can cause irreversible neurological and behavioural impairments^(2,3)^.

The WHO suggest that maternal iodine requirements are increased by 50 % during pregnancy and breast-feeding to meet the requirements of the growing fetus and feeding infant, respectively^(4)^. Recommended daily intakes of iodine are 250 µg for pregnant and lactating women, 150 µg for other adults and 90 µg/d for infants and young children (aged 0–59 months) to ensure both needs are met and that there is some thyroidal accumulation^(2)^. In the UK, the reference nutrient intake (RNI) for iodine is lower than the WHO recommendation, at 140 µg/d for all adults (no increment for pregnant and lactating women) and 60 µg/d for infants aged 4–12 months^(5)^. These guidelines assume that the UK is an area of iodine sufficiency, and that the iodine status of young women is sufficient to meet the demands of pregnancy and lactation^(5)^. The most recent National Diet and Nutrition Survey (NDNS) results for women of childbearing age (16–49 years), showed the median urinary iodine concentration (mUIC) was 98 µg/l, which is adequate. However, 21 % had a mUIC below 50 µg/l, which may be insufficient for some individuals^(6)^. This also falls significantly short of the WHO criterion for pregnant and lactating women (150–249 µg/l)^(2)^. Median iodine consumption was 124 µg/d for 19–64-year-old women, which is also below the WHO recommended intake or UK RNI^(2,5)^. Again, this may indicate some individuals have intakes which may be too low. Iodine intake data is not available separately for women of childbearing age (16–49 years). The diet and nutrition survey of infants and young children (DNSIYC) reported adequate iodine intakes of 168–176 µg/d (depending on ethnicity) in 2011, in infants aged 4–11 months, since then, no nationally representative data has been available^(7)^.

Without an iodised salt programme, the main dietary sources of iodine in the UK are milk and other dairy products, fish, and eggs^(8)^. An increase in plant-based diets, concern over the environmental impact and CO_2_ emissions associated with fish consumption and farming of eggs and dairy, have contributed to the re-emergence of ID in the UK^(9)^. Restriction of dairy product consumption is further promoted by the EAT-Lancet report, whilst the UK Eatwell guidance suggests reducing dairy and including plant-based milk-alternative drinks, alongside dairy products, which are not fortified with iodine by law^(10,11)^. Awareness of the importance of iodine and iodine-rich foods is poor in both younger women and their healthcare professionals (HCPs), particularly when compared with their general nutritional knowledge^(12)^. Further analysis of the UK NDNS survey data has shown that exclusive users of milk-alternative drinks have significantly lower iodine intakes (94 µg/d, *n* 3399) than conventional cows’ milk users (129 µg/d, *n* 88; *P* < 0·001)^(6)^. Vegans and those with an allergy to seafood, dairy or eggs are also at risk of ID^(13)^.

With the increasing popularity of plant-based diets and the move away from conventional milk and dairy products, it is easy for women of childbearing potential to become unwittingly iodine deficient with potential negative consequences for them and their children. The nutritional impact of complementary feeding practices on the micronutrient content of infant diets has also tended to focus on Fe, Zn and Na with little emphasis on iodine intakes^(14–16)^. Without the inclusion of iodine-rich foods or fortified infant formula and complementary foods, iodine intakes may be insufficient^(17)^. Given the importance of iodine for infant development, this study aimed to explore the iodine intake of mother and infant pairs in the UK, during the complementary feeding period (infants aged 6–12 months).

## Methods

A detailed description of the recruitment and data collection are provided elsewhere^(18)^. In brief, the study was cross-sectional and aimed to collect maternal and infant nutritional data as part of a study exploring complementary feeding practices. Participants were self-selecting caregivers of infants aged 6–12 months, recruited via advertisements placed on social media sites. Data were collected between 4 October 2019 and 1 December 2020^(18)^. A written explanation of the study was provided via the JISC survey platform,^(19)^ and participants were offered an email address and telephone number of the lead researcher if they wanted to discuss the study further. Participants consented by clicking ‘Yes – I have read the study information and consent to taking part in the study’ and completed an initial questionnaire online. Questions related to maternal demographic variables (such as age, occupation, education, parity, weight, height, special diets and allergies), infant characteristics (including birthweight, age, special diets and allergies) and infant age and the method of complementary food introduction and infant milk feeding history (breast and formula feeding). Participants were also asked (optionally) for a phone number, which was used by a researcher to complete one multi-pass 24-h recall, following a standardised methodology, for both caregiver and baby^(20)^. Participants were not made aware in advance, of when their 24-h recall would be completed. Collection and reporting of the dietary information relating to the infants in the study has been previously reported^(18)^. A requirement of the study was that caregivers were aged ≥ 18 years and resident in the UK.

### Nutritional analysis

Maternal 24-h recalls (foods and individual recipes) were entered into Nutritics^(21)^ by two researchers. All data entry was double checked by the lead researcher. Brands were entered where they were described by participants. Where brand names were provided but micronutrient data were missing in Nutritics (and not available on grocery or the manufacturer’s website), a food was selected which had micronutrient data that most closely matched the food on the 24-h recall, containing a similar energy and macronutrient composition. Where participants could not recall a brand or where brand information was missing, foods were chosen and entered according to a standard operating procedure to ensure consistency. New foods were inputted per 100 g using data from grocery (e.g. Tesco®, Sainsbury’s®) or manufacturer’s websites. This methodology aimed to minimise over- or under-reporting of iodine intake due to missing micronutrient data in Nutritics^(21)^. Portion size data (pack sizes, slices, estimated number of grams or ounces or household measures; tablespoons, teaspoons, cups and bowls) were provided by participants and entered directly into Nutritics. Where pack size information was missing, portion sizes were estimated using manufacturers websites. Where other portion size or brand information was missing, a medium or average portion size was assumed and estimated using Nutritics^(21)^ or the Food Portion Size handbook^(22)^. Brands were analysed according to the nutrient content available on Nutritics in June/July 2021. Some plant-based milk-alternative drink brands may have been fortified with iodine since data were collected or updated in Nutritics since data were entered and exported. Recipes were entered using the information provided by participants, including ingredients, preparation and cooking methods. Recipes were adjusted for nutrient losses, and weight change (water absorption or loss) during cooking before portion sizes were entered. Participants were asked if they had taken a vitamin, mineral or other supplement on the day of the recall, and to detail the brand. These were included in the analysis.

### Grouping foods for analysis

Foods were grouped according to type for the food group analysis. For example, ‘Fish’ included any fish or fish-based dish. ‘Eggs’ includes any egg or egg-based dish (including omelettes which may have contained other iodine containing foods such as cheese). ‘Yoghurt, Cream and Dairy desserts’ included dairy yoghurts, pancakes, custard, cheesecake, ice-cream, cream, milkshake and smoothies, ‘Non-dairy yoghurt & desserts’ included non-dairy yoghurt and ice-cream (no other non-dairy desserts or milkshakes were recorded). ‘Milk-alternative drinks’ included oat, soya, almond and coconut milks.

### Recommended Iodine Intake

The UK RNI for iodine in women is 140 µg/d and although the British Dietetic Association (BDA) and WHO suggest an increased intake during pregnancy and lactation (200 and 250 ug/d, respectfully), no official UK government recommendation exists. Iodine intakes were, therefore, compared with the UK government RNI of 140 ug/d for women of childbearing age.

### Calculations and statistical analysis

A simplified NS-SEC code^(23)^ was assigned to both the participant and their partner based on their occupation. These were combined and the highest occupation class used to classify each household.

Nutritional data and survey data were both exported to SPSS Statistics for Windows, version 24·0^(24)^ and checked for potential outliers. Tests for normality were carried out using Shapiro−Wilk and Kolmogorov−Smirnov tests. A Pearson’s correlation was used to explore correlation between continuous parametric data, whilst a Spearman’s rank-order correlation was used for continuous non-parametric data. *χ* ^2^ and Fishers exact tests were used on frequency data. An independent samples *t* test was used where data were continuous and parametric. Mann–Whitney-*U* tests were used where data were continuous or ordinal and non-parametric. A significance level of *P* < 0·05 was used throughout, except where a Bonferroni adjustment was applied where multiple correlations were used. Based on fifteen tests, the adjusted *P* value was *P* < 0·003.

## Results

### Maternal demographic characteristics

In total, 319 respondents completed the online survey, all of whom were the baby’s mother. Of the 189 respondents who left a phone number, 102 completed one 24-h recall. Of those who completed a recall with a researcher, eleven women were excluded from the analysis as their baby was aged over twelve months (three), born prematurely (two) or had an incomplete maternal recall (six). Ninety-one mother–infant pairs met the study criteria and were included in the analysis (Table 1).

**Table 1.** Maternal demographic characteristics. All participants and comparison between those who meet and do not meet RNI for iodine (food and food supplements)

	All (*n* 91)			≥ 140 µg/d iodine (*n* 46)		< 140 µg/d iodine (*n* 45)		*P* value (Chi-squared)
	Mean or frequency (*n*)		sd or %	Mean or frequency (*n*)	sd or %	Mean or frequency (*n*)	sd or %	
Age years (mean)	33·2		4·1	33·6	3·7	32·9	4·4	0·416†
Age category								0·121
18–25 years	1		1·1	0	0·0	1	2·2	
26–30 years	20		22·0	6	13·0	14	31·1	
31–35 years	51		56·0	31	67·4	20	44·4	
36–40 years	13		14·3	7	15·2	6	13·3	
> 40 years	6		6·6	2	4·3	4	8·9	
Status								
Single	5		5·5	1	2·2	4	8·9	
Cohabiting	14		15·4	9	19·6	5	11·1	0·231
Married	72		79·1	36	78·3	36	80·0	
Education								0·750
No formal/General Certificate of Secondary Education (GCSE)	2		2·2	1	2·2	1	2·2	
Further education	17		18·7	10	21·7	7	15·6	
Graduate/postgraduate	72		79·1	35	76·1	37	82·2	
Household social class								0·105
Higher managerial (I)	74		81·3	38	82·6	36	80·0	
Intermediate occupations (II)	11		12·1	6	13·0	5	11·1	
Routine/manual occupations (III)	4		4·4	0	0·0	4	8·9	
Unemployed/unwaged (IV)	2		2·2	2	4·3	0	0·0	
Singleton birth	90		98·9	46	100	44	97·8	0·495‡
Primiparous	54		59·3	24	52·2			0·202‡
Ethnicity								0·767
White British	77	84·6		40	87·0	37	82·2	
Other White	6	6·6		2	4·3	4	8·9	
Black/Black British	1	1·1		0	0·0	1	2·2	
Asian/Asian British	4	4·4		2	4·3	2	4·4	
Mixed Race	3	3·3		2	4·3	1	2·2	
Breastfeeding								0·025*
Breast milk only	50		54·9	30	65·2	20	44·4	
Formula milk only	26		28·6	13	28·3	13	28·9	
Mixed feeding (breast and formula milk)	15		16·5	3	6·5	12	26·7	
Self-reported maternal dairy allergy	4		4·4	1	2·2	3	6·7	0·299
Taking supplements	42		46·2	28	60·9	14	31·1	0·004*
Taking supplements with iodine	17		18·7	17	37·0	0	0·0	< 0·001*

RNI, reference nutrient intake.

* *P* value < 0·050 indicates significance.

† Mann–Whitney *U* test.

‡ Fisher’s exact test.

The mean age of the women was 33·2 years (sd 4·1 years). Most women included in the study were exclusively breast-feeding (54·9 %) with a smaller proportion formula feeding (28·6 %) or mixed feeding (16·5 %). Most of the mothers in this study were married (79·1 %) and highly educated (79·1 % graduate/postgraduate level education) with 81·3 % employed in higher management roles.

### Infant characteristics

The mean age (sd) of babies was 8·4 (1·3) months, and mean birthweight was 3·5 kg. Seventy-one percent of babies were being breastfed some breast milk at 6 months of age and the majority did not follow dietary restriction (91·2 %).

### Maternal iodine intake

The estimated total maternal iodine intake from food and supplements (median + IQR) met the UK RNI for women 140·3 (11·2–151·5 µg/d) (Table 3). Estimated total median iodine intake of babies from food and formula or breast milk exceeded the RNI (60 µg/d) at 96·9 (34·6–159·2 µg/d). Sixty-one percent of the estimated baby iodine intake (median + IQR) was from breast milk (Table 3).

In this study, 49·5 % of mothers did not meet the RNI for iodine compared with 50·5 % of mothers who did. There was no significant difference in the age of mothers who met the RNI for iodine (≥ 140 ug/d) and those who did not (< 140 ug/d) (Table 1). A significantly higher proportion of mothers who met the RNI for iodine complemented their diet with supplements (60·9 %) compared with those who did not meet the RNI for iodine (31·1 %, *P* = 0·004). Likewise, a significantly greater proportion of mothers who met the RNI for iodine complemented their diet specifically with iodine containing supplements (37·0 %) compared with those who did not meet the RNI for iodine (0·0 %, *P* < 0·001).

There was no significant difference in age, birthweight, feeding practices or the age at which solid foods were introduced between babies with mothers who met the RNI for iodine and those who did not (Table 2).

**Table 2.** Infant characteristics overall, and by whether maternal iodine intakes meet or do not meet RNI for iodine

	All (*n* 91)		≥ 140 µg/d (*n* 46)		< 140 µg/d (*n* 45)		*P* value (Fisher’s Exact)
	Mean/Frequency (*n*)	sd or %	Mean/ Frequency (*n*)	sd or %	Mean/ Frequency (*n*)	sd or %	
Baby age (months)	8·4	1·3	8·6	1·3	8·3	1·3	0·288*
Baby age category							0·533†
6–8·5 months	56	61·5	28	60·9	28	62·2	
9–12 months	35	38·5	18	39·1	17	37·8	
Birthweight (kg)	3·5	0·5	3·5	0·5	3·5	0·5	0·661*
Age solids introduced (weeks)‡	24·1	2·3	24·9	1·8	23·6	2·4	0·052*
Ever breastfed	88	97·0	44	95·7	44	97·8	0·508
Breastfed ≥ 26 weeks	71	78·0	37	80·4	34	75·6	0·449
Currently breast fed	65	71·4	33	71·7	32	71·1	0·566
Self-reported dairy allergy	10	11·4	5	11·1	5	11·6	0·601
Vegan	1	1·1	0	0·0	1	2·2	0·495
Vegetarian	2	2·2	2	4·3	0	0·0	0·495
Pescatarian	5	5·5	4	8·7	1	2·2	0·187
No restriction	83	91·2	40	87·0	43	95·6	0·267
Self-reported infant dairy allergy	10	11·0	5	10·9	5	11·1	0·939
Baby-led weaning style§	33	36·3	16	34·8	17	37·8	0·468
Supplement	26	28·6	15	32·6	11	24·4	0·265

* Mann–Whitney *U* test.

† *χ* ^2^ test.

‡ *n* 49 (19 participants who met Scientific Advisory Committee on Nutrition (SACN) iodine requirement and 30 who did not), as question was missing from 1 questionnaire.

§ Infants following baby-led weaning are being spoon fed ‘10 % of the time or less’ and are also ‘receiving purees 10 % of the time or less’, as self-reported by parents.

### Maternal dietary iodine from food sources

**Figure 1.**
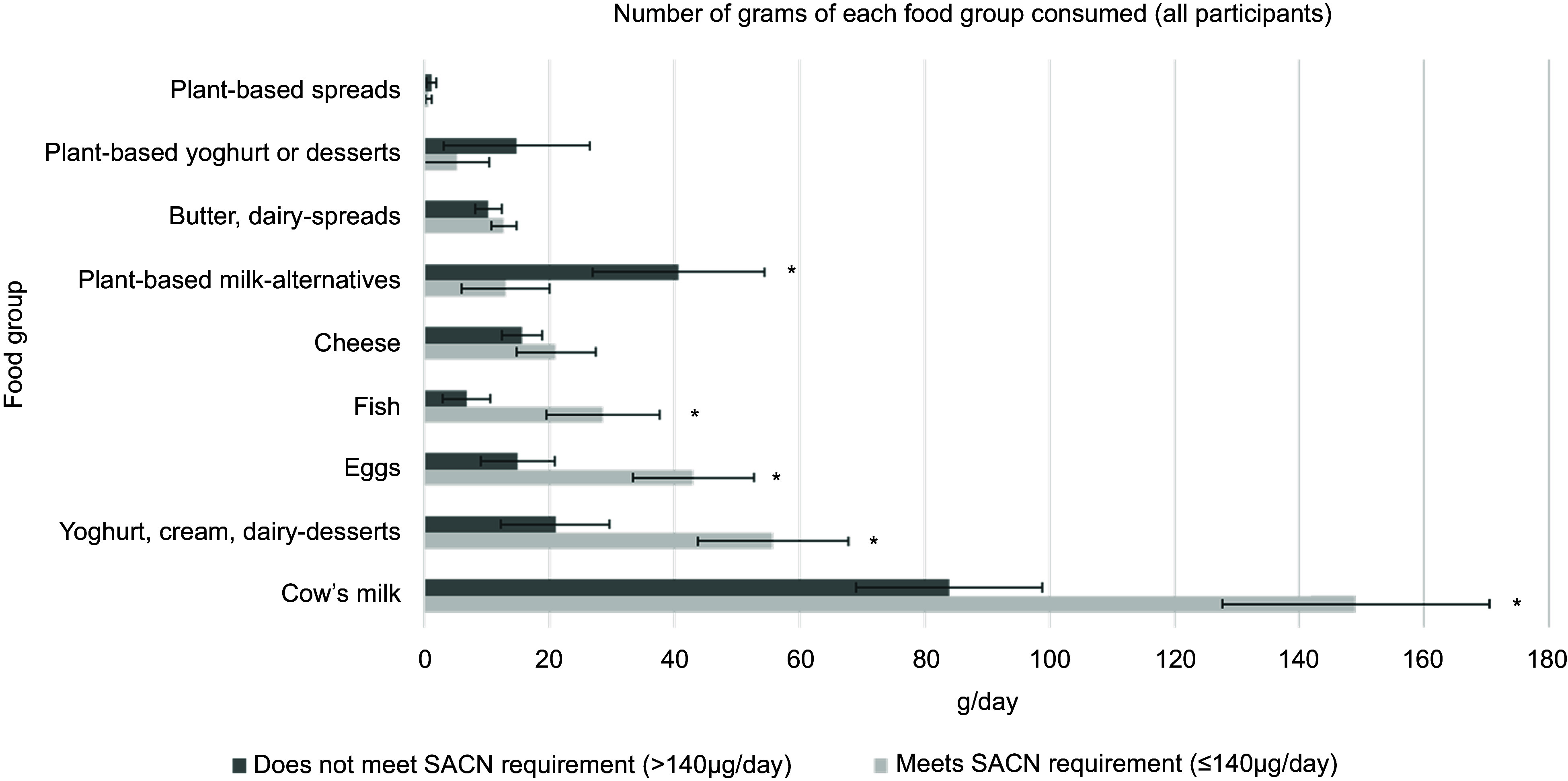
Comparison of estimated maternal intake (using *χ* ^2^) of commonly consumed iodine-rich foods, dairy products and plant-based milk-alternatives (g/d and se of the mean), between those who meet iodine requirements (≥ 140 ug/d) and those who do not (< 140 µg/d). * Denotes a significant difference between groups (*t* test, *P* < 0·05).

Mean intakes (g/d) of commonly consumed iodine food sources and plant-based milk-alternative drinks in mothers who met the UK RNI for women (≥ 140 g/d) and those who did not (< 140 g/d) were estimated (Figure 1). Estimated intakes of fish, eggs, cow’s milk and yoghurt/cream/dairy desserts were significantly greater in mothers who met iodine requirements compared with those who did not (*P* < 0·05). No significant difference was observed between groups for cheese and butter/dairy spread intake (*P* > 0·05). Estimated intake of plant-based milk-alternative drinks was significantly greater in mothers who did not meet recommended iodine intakes compared to those who did (*P* < 0·05). No significant difference was observed between groups for intakes of non-dairy spreads and non-dairy yoghurts/desserts.

### Maternal energy intake

**Figure 2.**
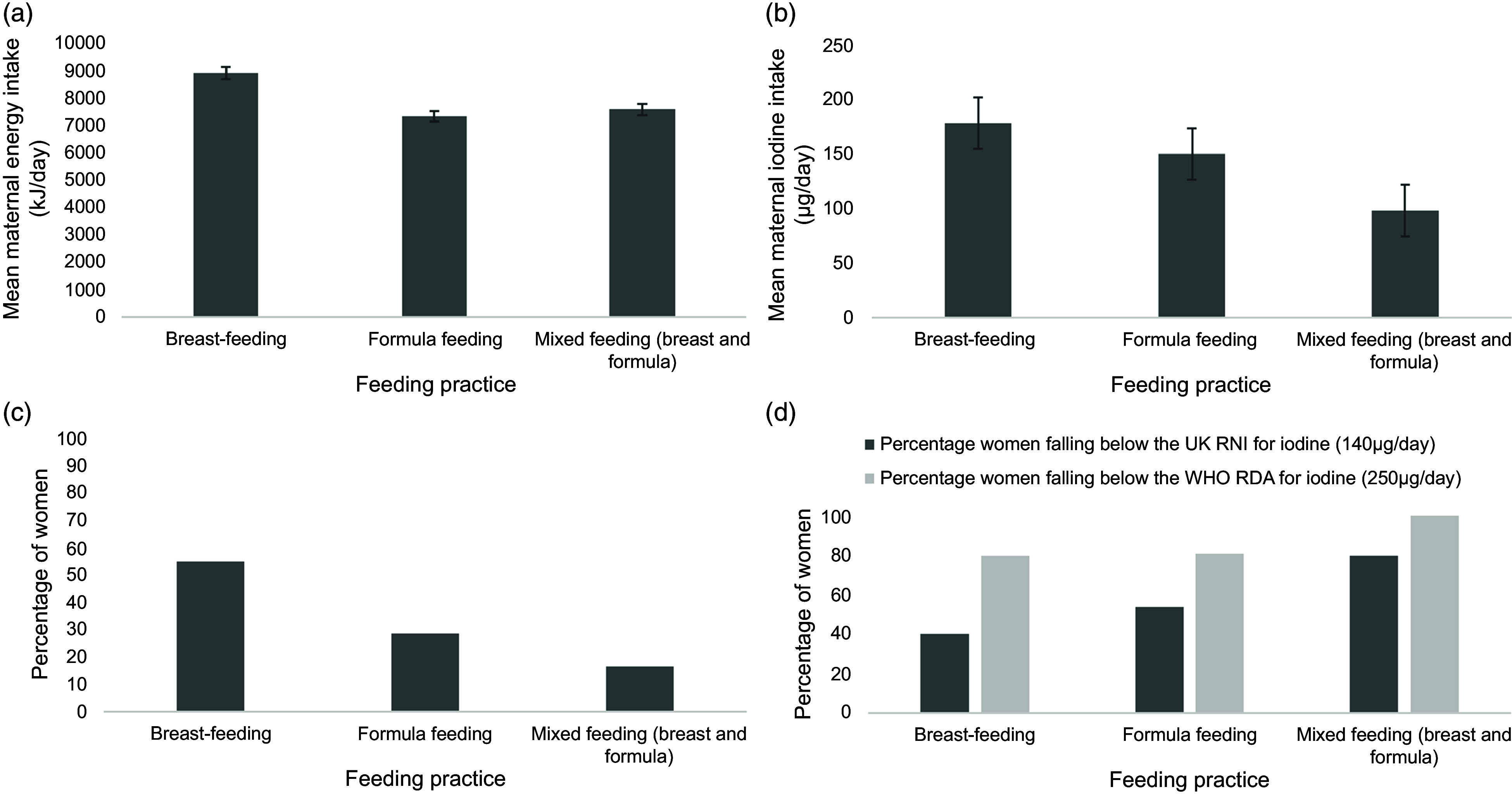
(a) Estimated maternal energy intake (kJ/d) of women grouped according to feeding practice (mean + sem). (b) Mean maternal iodine intake amongst women, by feeding practice. (c) Percentage of women exclusively using breast milk, exclusively using formula milk or a mix of breast and formula to feed their infants. (d) Percentage women falling below the UK RNI for iodine (140 µg/d) and WHO RDA for iodine (250 µg/d). RNI, reference nutrient intake.

Estimated daily energy intake did not differ between mothers who met the recommended iodine intake for lactating women mean (sd) 8694 kJ (1941 kJ) and those who did not 7736 kJ (2235 kJ) (Figure 2). However, breast-feeding women reported significantly greater estimated daily energy intake (kJ) compared with women feeding their babies formula and a mixed approach (breast-feeding and formula) (*P* < 0·05). Mean maternal energy intake differed depending on feeding practice. An ANOVA and Fisher’s Least Significant Difference (LSD) post hoc test showed breast-feeding women consumed significantly more energy (8878 kJ) than women who were formula feeding (7300 kJ) but not more than women who were mixed feeding (7556) (*P* = 0·002) (Figure 1(a)).

### Maternal iodine intake and infant iodine intake

Maternal total energy intake (kJ/d) was negatively associated with infant total iodine intake (µg/d) and infant iodine intake from breast or formula milk (µg/d, *P* < 0·05) (Table 4) but not following a Bonferroni adjustment (based on a *P* value of *P* < 0·003). However, total maternal iodine intake, maternal iodine intake from food only and maternal iodine intake from dairy foods only were significantly associated with increased infant iodine intake from food (*P* < 0·05) but not following a similar Bonferroni adjustment (*P* < 0·003).

**Table 3.** Energy and iodine intake of mothers and babies in the sample, from food, milk and food/milk combined

	Maternal			Baby		
	Food	Supplement	Total	Food	Milk* (breast, formula or both)	Total
Energy						
Mean	8206	4	8210	1952	1868	3819
sd	2140	20	2138	864	417	732
Range	3010–12 615	0–172	3010–12 615	404·4–4796·0	390·2–2945·7	2437–5477
Median	7831	0	–	1892	1854	3845
IQR	4751–10 911	0	–	581·9–3202·1	1488·2–2219·8	2727·7–4962·3
Iodine (µg)						
Mean (sd)	130·5	29·3	159·8	40·3	61·7	102·1
sd	74	64·4	103·0	35·8	29·0	41·2
Range	24·0–397·0	0·0–300·0	24·0–547·0	1·0–161·1	20·4–163·3	46·1–216·4
Median	117·3	0·0	140·3	29·1	49·6	96·9
IQR	13·8–220·8	0·0–0·0	11·2–151·5	−20·5–78·7	18·9–80·3	34·6–159·2

IQR, interquartile range.

* Breast milk intake was estimated in breast/mixed-fed infants^(21)^.

**Table 4.** Spearman’s correlation coefficient demonstrating the association between maternal iodine intake and infant iodine intake

	Infant total iodine intake (µg)	Infant iodine intake (food only) (µg)	Infant iodine intake (milk only) (µg)
Maternal total energy intake (kJ)	−0·225*	−0·066	−0·240*
Maternal total iodine intake (µg)	0·139	0·243*	−0·159
Maternal iodine intake (supplements only) (µg)	0·101	0·074	0·010
Maternal iodine intake (food only) (µg)	0·132	0·238*	−0·160
Maternal iodine intake (dairy foods only) (µg)	0·189	0·264*	−0·480

* *P* ≤ 0·05, Spearman’s rho.

*P* ≤ 0·003, Spearman’s rho with Bonferroni adjustment (no values were significant).

Mean maternal iodine intake also differed between groups. An ANOVA with LSD post hoc test demonstrated women who were breast-feeding had a greater intake (179 µg/d) compared with those mixed feeding (99 µg/d) but not compared to those formula feeding (150 µg/d) (*P* = 0·007) (Figure 2(b)). A chi-squared test comparing the number of women meeting/not meeting UK RNI (140 µg/d) by feeding type showed a significant difference between groups (*P* = 0·019) but there was no difference in the number of women meeting or not meeting the WHO Recommended Daily Amount (RDA) for iodine (250 µg/d) (Figure 2(d)).

## Discussion

In this study, total iodine intake was greater than that reported in the UK NDNS (median 124 μg/d) for women aged 18–64 years, although pregnant and lactating women were excluded from the NDNS^(6)^. Breast-feeding women are likely to have higher iodine intakes, as energy requirements are higher, and they are likely to consume more food than women who are not pregnant or lactating. Indeed, median total iodine intake was higher in breast-feeding mothers, compared to non-breast-feeding mothers in this study, but intakes were lower than both the WHO recommendation of 250 µg/d for lactating women^(2)^ and the BDA recommendation of 200 µg/d^(25)^. These are population level guidelines, and would exceed the requirement of most individuals, however, amongst women exclusively breast-feeding, 16 % were also not meeting the UK Lower Reference Nutrient Intake (LRNI) of 70 µg/d, the estimated dietary intake of iodine required to avoid goitre manifestation^(26)^.

The concentration of iodine in breast milk is affected by maternal iodine intake and diminishes over time,^(27,28)^ whilst the mUIC of infants, is positively correlated with their mother’s breast milk iodine concentration^(29)^. If the iodine intake estimated from the single 24-h dietary recalls in this study is representative of the mothers’ average iodine intake, then the iodine content of breast milk may be insufficient to meet infant requirements. This cannot be known, however, without taking samples of breast milk and assessing the iodine status of both mothers and infants via mUIC. Furthermore, iodine may be partitioned into breast milk, rather than urine when intake is low, protecting infants from deficiency^(30)^. Worldwide, there has been a steady increase in the number of countries that have adequate population-level iodine intake, with 57 % of countries rated as sufficient in 2022^(31)^. Unlike many countries, however, the UK has no fortification programme and has seen a downward trend in iodine intake over the past decade^(32)^.

The median total infant iodine intake (from food and breast or formula milk feeds) was comparable to that previously published by Fallah *et al.* (2019) who estimated iodine intake to be 89 µg/d) in a cohort of US infants of the same age. In the present study, 18·7 % of infants were not meeting UK RNI for iodine (60 µg/d), but no infant was below the LRNI of 40 µg/d^(33)^. It should be noted, however, that breast milk intake was estimated (1) based on the age of the baby, using previously published data^(33)^ and (2) calculated, reliant on published data on iodine content of human breast milk^(21)^.

Government guidelines in the UK do not currently recommend iodine supplementation and women are not screened for iodine insufficiency during pregnancy,^(26)^ but a few studies exploring first trimester iodine status in the UK has found levels to be insufficient^(9)^. A study found few women (12 %) received information about iodine during their pregnancy and only 6–9 % recognised different dairy products as being sources of iodine^(12)^. Despite this almost 20 % of the study participants took a supplement containing iodine on the day of their dietary recall and those who supplemented with iodine, also had higher intakes of iodine from food sources. This could be due to awareness or just general health consciousness, whereby women were taking a supplement and also choosing a nutrient-dense and balanced diet to support feeding their baby. Not all breast-feeding women met requirements, however, suggesting awareness of iodine-rich foods and supplementation should be part of public health guidance for pregnancy and lactation. The fortification of foods such as salt or plant-based milk-alternative drinks, which are mandatory in other countries, should also be considered in the UK, to support those who do not eat seafood or dairy products. Kirk *et al.* (1999) found vegetarians, vegans or pescatarians were more likely to supplement their diet, as were those with a greater number of positive health behaviours, such as consuming more fruit and vegetables, being more physically active, maintaining a BMI in the healthy range or having a lower alcohol intake,^(34)^ but a systematic review by Eveleigh *et al.* (2023) found vegan diets to be insufficient in iodine intake and were associated with lower iodine intakes compared with omnivorous diets (*P* < 0·001)^(13,35)^. As vegans are more likely to breastfeed than vegetarians or non-vegetarian/vegans this could also result in iodine insufficiency for both mother and infant^(36)^. Our study observed few vegans, vegetarians or pescatarians, but many individuals used plant-based milk-alternative drinks, possibly due to allergy, cow’s milk protein allergy in their infant, as part of a flexitarian diet or as a move towards more sustainable plant-based diets. Plant-based milk-alternative drink consumption was significantly higher in those who did not meet the RNI for iodine and it could be that non-vegan participants were consuming plant-based milk-alternative drinks, without considering the impact on iodine intake.

In this study, self-reported dairy allergy (cow’s milk protein allergy) amongst infants was high (11 %) but was not associated with an increased likelihood of iodine intake below the RNI. Breast-feeding mothers who have a baby with cow’s milk protein allergy are advised to eliminate cow’s milk and other dairy products from their diet for 6 weeks, to see if the baby’s symptoms improve^(37)^.

Furthermore, consumer data show 30 % of all consumers and 40 % of consumers with a child aged under 4 years in their household, consumed plant-based milk-alternative drinks^(38)^. Research suggests that plant-based milk-alternatives are typically lower in iodine compared with dairy milk (0·36 + 0·08 mg kg –1 and 0·067 + 0·109 mg kg^–1^, respectively^(39)^ and that iodine levels in the plant-based milk-alternatives show greater variability due to inconsistent fortification^(39)^. This further emphasises the need to make fortification of plant-based milk-alternative drinks mandatory for those who are unaware of the need for sufficient iodine intake. This may help to increase the iodine content of breast milk amongst those avoiding dairy due to allergy of themselves or their baby.

Women who met the RNI for iodine consumed more cow’s milk, other dairy products, eggs, and fish. Although correlations between maternal iodine intake and infant iodine intake were NS after a Bonferroni correction, if babies are sharing in family mealtimes and eating similar foods to their parents, this could further highlight that a nutritionally adequate maternal diet translates to a better-quality infant diet, consistent with studies that highlight the positive influence of maternal diet on infant eating behaviours^(40)^. Higher maternal energy intake showed a significant negative correlation with total infant iodine intake and infant iodine content from breast or formula milk, but this effect also disappeared following a Bonferroni adjustment. An association would be challenging to explain but could demonstrate underestimation of the amount of breast milk being consumed by the baby. Alternatively, women with higher energy intakes may be consuming more energy dense foods which are also high in sugar, and which would not be shared with their baby.

It is important to recognise the limitations of this study. The study was small, women were largely white British, well-educated and from higher socio-economic groups and almost 80 % of women had a degree or postgraduate degree, compared with 39 % of working-age people nationally^(41)^. Previous studies have demonstrated that women of higher socio-economic status or with more years in education are more likely to afford or chose a diet which is sufficient, and the data may not be comparable to a group of women with a lower income^(42)^. The iodine content of food varies greatly depending on the country, soil where it was produced, farming practices and food or safety regulation^(25)^. In this study, 71·4 % of women were offering their baby breast milk, compared with <1 % nationally, when babies were 12 months of age^(43)^. A high proportion of women excluded dairy products on the day of measurement, suggesting study participants may be more health conscious or concerned about their diet and health, when compared with the general population. Where dairy products are purposefully avoided, higher socio-economic groups could be more likely to afford plant-based milk-alternative drinks which are fortified, questioning the generalisability of the findings. Veganism does not always result in a healthy diet, however, with many vegans basing their meals on convenience foods^(44)^. Furthermore, in this current study, nutritional data were collected via one 24-h recall. Whilst useful for large studies, quickly administered and sensitive to a broad range of diets, 24-h recall is known to underestimate total energy intake in adults by an average of 11 % with up to 21 % underreporting amongst obese women^(45)^. Energy intake in infants, meanwhile, is likely to be over-estimated, especially when a wider range of foods are consumed across the day. This may be due to the accrual of small overestimates in portion size and underestimates in food spat out or dropped, for each food item consumed^(46)^. Intakes of breast milk are a further source of potential inaccuracy over estimating iodine intake in infants, although in this study, this was based on ‘average intake for age’ which has less overestimation than ‘time spent feeding’^(47)^. This introduces uncertainty into the results as the volume of breast milk consumed during complementary feeding is highly variable and will depend on factors other than age and may limit the accuracy of the results. Results would be different if the EAR or WHO cut-offs were used. Data were entered in 2021, since when some brands of milk-alternative drinks may have been fortified and Nutritics may have been updated with iodine data after the study data was entered^(48)^. Caution should be used when generalising these findings to countries outside of the UK, where foods may be fortified with iodine.

### Conclusion

This study adds to a body of evidence suggesting women in the UK may not consume enough iodine to meet the demands of pregnancy and lactation. Appropriate guidance on iodine-containing foods, a greater understanding of iodine intake before and during pregnancy and lactation, mandatory fortification of plant-based milk-alternatives and consideration of mandatory salt iodisation for home cooking could all serve to reduce the risk of ID amongst women and children in the UK. Further consideration of UK iodine intake RNI’s for pregnant and lactating women is required.
